# Non-HDL cholesterol and LDL cholesterol in the dyslipidemic classification in patients with nonalcoholic fatty liver disease

**DOI:** 10.1186/s12944-017-0621-4

**Published:** 2017-12-02

**Authors:** Tingting Du, Xingxing Sun, Xuefeng Yu

**Affiliations:** 1 Department of Endocrinology, Tongji Hospital, Tongji Medical College, Huazhong University of Science and Technology, Wuhan, 430030 China; 2 Department of Anesthesiology, Tongji Hospital, Tongji Medical College, Huazhong University of Science and Technology, Wuhan, 430030 China

**Keywords:** Nonalcoholic fatty liver disease, LDL-cholesterol, Non-HDL-cholesterol

## Abstract

**Background:**

Low-density lipoprotein cholesterol (LDL-C) always underestimates the true cholesterol burden in patients with nonalcoholic fatty liver disease (NAFLD). We aimed to compare LDL-C and non-high-density lipoprotein cholesterol (non-HDL-C) in the identification of high-risk dyslipidemic phenotypes in those with NAFLD.

**Methods:**

We conducted a cross-sectional analysis using a cohort of 9560 apparently healthy Chinese adults who underwent comprehensive health checkups including abdominal ultrasonography.

**Results:**

Among 3709 patients with NAFLD, the prevalence of abnormal LDL using LDL-C was 68.5%, whereas the prevalence was relatively lower when using non-HDL-C (55.9%). The concordance between non-HDL-C- and LDL-C-based diagnoses of abnormal LDL was similar in the hypertriglyceridemic (ҝ = 0.56; 95% CI 0.52–0.60) and normotriglyceridemic subgroups (ҝ = 0.47; 95% CI 0.44–0.51). Non-HDL-C detected fewer patients with abnormal LDL than LDL-C in normotriglyceridemic patients. However, non-HDL-C detected more patients with abnormal LDL than LDL-C in hypertriglyceridemic patients: 114 of the 1662 patients considered as abnormal LDL according to LDL-C fell into the normonon-HDL-C phenotype, whereas 204 of the 1662 patients considered as abnormal LDL according to non-HDL-C fell into the normoLDL-C phenotype.

**Conclusion:**

Among patients with NAFLD, LDL-C is superior to non-HDL-C in the detection of high-risk phenotypes in normotriglyceridemic patients, whereas non-HDL-C seems to be superior in hypertriglyceridemic patients.

## Background

The worldwide prevalence of nonalcoholic fatty liver disease (NAFLD) is increasing rapidly, affecting between 15%–40% of adults [1, 2]. Dyslipidemia that frequently coexist with NAFLD [3] has been identified as a major modifiable risk factor for the accelerated development of cardiovascular disease (CVD) [4]. Although no licensed pharmacological lipid-lowering strategy in patients with NAFLD exists, it is widely accepted that the lipid-lowering strategies for NAFLD and CVD are similar, aimed primarily at reducing low-density lipoprotein cholesterol (LDL-C) [5, 6]. Nevertheless, the major features of dyslipidemia in patients with NAFLD are an atherogenic lipid profile, consisting of high triglyceride (TG) levels, low high-density lipoprotein cholesterol (HDL-C), and an increase in TG-rich lipoproteins (including very-low-density lipoprotein [VLDL] and intermediate-density lipoprotein [IDL]), and small dense LDL particles) [3]. LDL-C concentrations have generally been reported to be at normal levels in the setting of NAFLD [7, 8]. Thus, LDL-C underestimates the true cholesterol burden in NAFLD as its concentrations do not fully capture the whole mass of lipoprotein particles [7]. Non-high-density lipoprotein cholesterol (non-HDL-C) represents a composite measure that encompasses the total cholesterol content within all VLDL, IDL and small dense LDL particles. A growing body of evidence has highlighted that non-HDL-C levels are at least moderately increased in NAFLD [8–10]. Furthermore, a recent study has demonstrated that non-HDL-C is stronger than other lipoproteins in predicting the onset of NAFLD [9]. In addition, emerging data have indicated that non-HDL-C is a better predictor of CVD than LDL-C [11, 12]. Therefore, measuring non-HDL-C may better identify lipid abnormalities in patients with NAFLD. However, no comparison has been made between non-HDL-C and LDL-C in the classification of patients with NAFLD into dyslipidemic phenotypes. Hence, we aimed to compare the classification of a group of patients with NAFLD into dyslipidemic phenotypes using non-HDL-C and LDL-C.

## Methods

### Study population

The study participants were Chinese employees and retired workers aged 20–100 years from the Wuhan Iron and Steel Company (WISCO), which is one of the largest iron and steel companies in China. Full details of the study have been described elsewhere [13]. The present cohort included employees and retired workers free of known CVD who received a comprehensive health examination (including abdominal ultrasonography) at the Healthcare system, WISCO general Hospital, between June 2008 and December 2010 (*n* = 15,199).

**Fig. 1:**
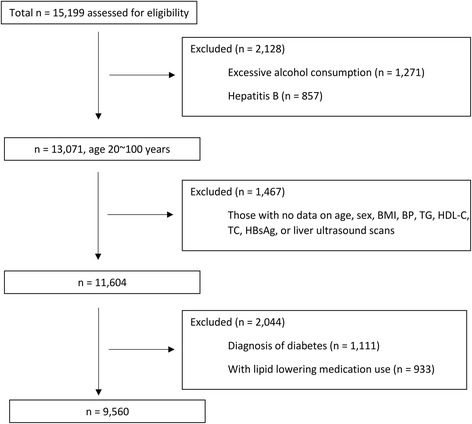
Flow diagram of subject inclusion and exclusion

All subjects were asked to complete a standard questionnaire that gathered information on alcohol consumption habits, histories of current and previous illness, and medical treatments. We excluded 5639 participants from this study, comprising 1271 with alcohol consumption in amounts >70 g/week for women (73) and >140 g/week for men (1198), 857 participants with hepatitis B surface antigen (HBsAg) positivity, and 1467 missing information on age, sex, anthropometric assessment, lipid measurements, test results for HBsAg, or liver ultrasound scans. In addition, to avoid the effects of lipid-lowering on all lipid parameters, 933 participants with lipid-lowering medication use were excluded. Furthermore, 1111 individuals with diabetes (defined using the 2015 American Diabetes Association (ADA) criteria [14] of a fasting plasma glucose ≥126 mg/dl or taking anti-diabetic medications for diabetes) were also excluded as diabetes has a strong independent relationship with increased levels of TG, decreased levels of HDL-C, and increased risk of NAFLD. The remaining available 9560 participants (6022 men and 3538 women) were included in our data analysis (Fig. 1). The fact that men accounted for 63% of total participants was in consistent with the proportion of male employees at WISCO. According to the Private Information Protection Law, information that might identify subjects was safeguarded by the Health Examination Center. This study was approved by the institutional review board of WISCO general Hospital. Because we only retrospectively accessed a de-identified database for purposes of analysis, informed consent requirement was exempted by the institutional review board. The procedures followed were in accordance with the ethical standards of the responsible committee on human experimentation and with the Helsinki declaration of 1975, as revised in 1983.

### Anthropometric and biochemical measurements

Anthropometric measurements, including weight, height, and systolic/diastolic blood pressure (BP) were measured following standardized protocols from the World Health Organization (WHO). Body mass index (BMI) was calculated as weight (in kilograms) divided by the square of height (in meters). Participants’ seated BP was measured twice for every 5 min on the right arm after 5 min of rest by trained nurses with a sphygmomanometer. The mean of the two readings was used in data analysis.

Overnight fasting (at least 8 h) blood samples were collected from the antecubital vein of each individual. Biochemical measurements, including assessment of fasting plasma glucose, total cholesterol (TC), TG, LDL-C, HDL-C, alanine aminotransferase, uric acid, and hepatitis viral antigen/antibody, were measured enzymatically on an autoanalyzer (Hitachi 7600, Ltd., Tokyo, Japan). Non-HDL-C was calculated as TC minus HDL-C. All the blood measurements were followed the same protocol.

### Assessment of NAFLD

Ultrasound tests were performed by trained sonographers using a high-resolution, real-time scanner (model SSD-2000; Aloka Co., Ltd., Tokyo Japan). One experienced radiologists used standard criteria in evaluating the images for the presence or absence of hepatic fat [15]. Generally, the diagnoses of fatty liver was based on the presence of stronger echoes in the hepatic parenchyma compared with echoes in the kidney or spleen parenchyma [16].

### Definitions

According to the current Adult Treatment Panel III of the National Cholesterol Education Program (NECP/ATP III) guidelines [17], elevated TG is defined as ≥150 mg/dl.

According to the ADA and the American College of Cardiology Foundation (ACC) report [18], elevated non-HDL-C is defined as ≥130 mg/dl.

### Statistical analysis

All statistical analyses were performed using SPSS software (version 12.0 for windows; SPSS, Chicago, IL, USA). Continuous variables were presented as medians and interquartile ranges (IQR) due to their skewed distribution. Categorical variables were presented as percentages. Kruskal-Wallis analysis of median test was used followed by the Mann-Whitney U test for pairwise comparisons. Bonferroni correction was applied to adjust *P*-values for multiple comparisons. Chi-square test was performed to assess differences in proportions across groups. Of the 9560 participants studied, 3709 patients were identified as NAFLD. The NAFLD patients were divided into four mutually exclusive groups by the presence or absence of TG ≥150 mg/dl and non-HDL-C ≥ 130 mg/dl. For comparison, patients were also categorized by the conventional approach based on TG and LDL-C cut points. For these analyses, levels of 150 mg/dl for TG and 100 mg/dl for LDL-C were chosen. LDL-C values corresponding to non-HDL-C concentrations are not available, a value of 100 mg/dl for LDL-C was chosen for identification of patients as dyslipidemic phenotypes per consensus report from the ADA/ACC panel [18]. Spearman correlation was adopted to assess coefficients between non-HDL-C and LDL-C. The kappa (ҝ) statistic was calculated to test for an agreement between non-HDL-C- and LDL-C-based identification of dyslipidemic phenotypes. Values for ҝ value can be between 0 and 1, with a value of ≥0.75 signifies perfect agreement, whereas with a value of <0.40 indicating poor agreement. Venn diagram was constructed as a visual display of concordance/discordance among TG-, LDL-C and non-HDL-C-based classification of participants. Significance was accepted at a two-tailed *p* < 0.05.

## Results

**Fig. 2:**
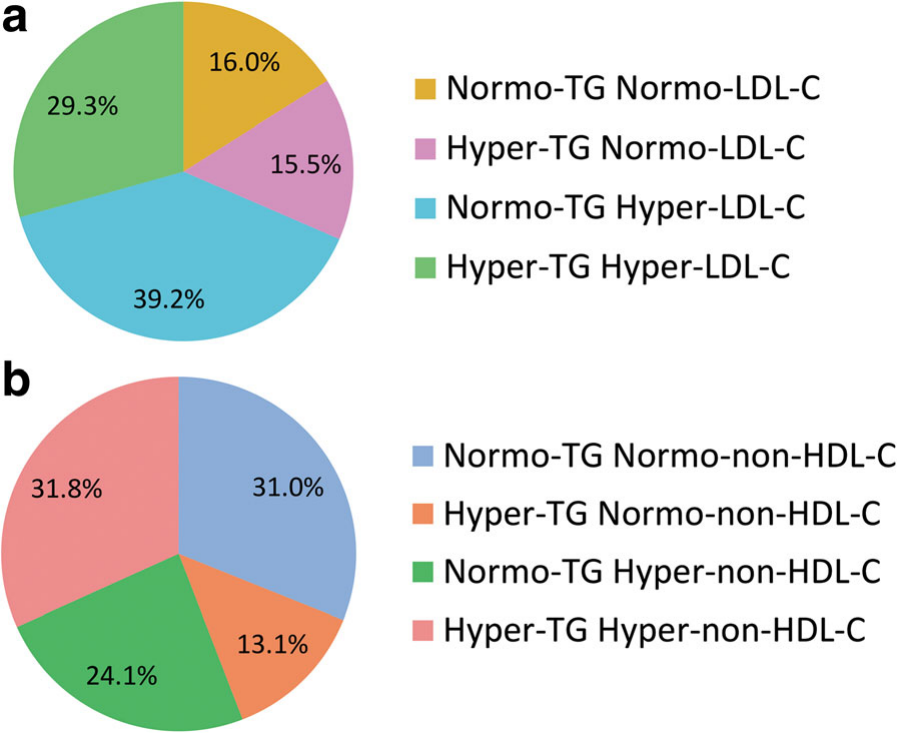
Lipid phenotype distributions of the 3709 patients with nonalcoholic fatty liver disease according to triglycerides and LDL cholesterol levels (**a**), triglycerides and non-HDL-cholesterol levels (**b**)

Characteristics of subjects with and without NAFLD were described elsewhere [19]. Using the conventional classification, 16.0% were identified as normal, 15.5% as hypertriglyceride-normoLDL-C, 39.2% as normoTG-increased LDL-C, and 29.3% as hypertriglyceride-increased LDL-C (Fig. 2a). Hence, 68.5% had abnormal LDL, as evidenced by increased LDL-C. When using TG and non-HDL-C to identify dyslipidemic phenotypes, 31.0% were identified as normal, 13.1% as hypertriglyceridemic-normonon-HDL-C, 24.1% as normoTG-hyper-non-HDL-C, and 31.8% as hypertriglyceridemic-hyper-non-HDL-C. (Fig. 2b). In total, 55.9% of the patients with NAFLD had abnormal LDL particle number and therefore abnormal LDL, as evidenced by increased non-HDL-C.

The characteristics of the four groups in Fig. 2a were showed in Table 1. Individuals with dislipidemia, irrespective of hypertriglyceridemia or increased LDL-C or by both, had higher levels of systolic BP, and manifested more worse lipid profile. Table 2 showed the characteristics of the four groups in Fig. 2b. Individuals with dislipidemia, irrespective of hypertriglyceridemia or increased non-HDL-C or by both, had higher levels of systolic BP and alanine aminotransferase, and manifested more worse lipid profile.

**Table 1:** Characteristics of participants by triglycerides (TG) and LDL-cholesterol status

	Normal-TG normal-LDL-C	Hyper-TG normal-LDL-C	Normal-TG hyper-LDL-C	Hyper-TG hyper-LDL-C
n	595	574	1452	1088
Age (years)	49.0 (41.0–56.0)	53.0 (45.0–61.0) ^*^	51.0 (42.0–58.0)^*†^	49.0 (41.0–58.0)^‡^
Body mass index (kg/m^2^)	25.5 (24.1–27.4)	25.5 (24.0–27.3)	26.0 (24.3–27.7)	25.1 (23.5–27.0)^‡^
Systolic blood pressure (mmHg)	120.0 (114.0–131.0)	127.0 (120.0–138.0) ^*^	130.0 (120.0–140.0) ^*^	129.0 (120.0–138.0) ^*^
Diastolic blood pressure (mmHg)	80.0 (75.0–90.0)	80.0 (74.0–90.0)	80.0 (76.0–90.0)	80.0 (70.0–85.0)
Fasting plasma glucose(mmol/l)	5.2 (4.8–5.6)	5.2 (4.8–5.6)	5.2 (4.8–5.6)	5.1 (4.7–5.5)
Uric acid (mmol/l)	5.4 (4.6–6.3)	6.0 (5.3–6.9) ^*^	5.9 (5.1–6.8) ^*^	5.4 (4.6–6.2)^†‡^
Alanine aminotransferase (U/L)	22.0 (16.0–32.0)	24.0 (17.0–33.0)	29.0 (20.0–43.0) ^*†^	29.0 (20.0–40.0) ^*†^
Total cholesterol (mg/dl)	148.3 (135.5–158.3)	187.6 (173.0–206.9) ^*^	204.4 (186.1–226.6) ^*†^	164.9 (148.6–183.0) ^*†‡^
Triglycerides (mg/dl)	93.8 (67.3–117.7)	107.1 (85.0–126.5) ^*^	202.7 (173.5–254.4) ^*†^	252.2 (194.7–372.6) ^*†‡^
LDL-cholesterol (mg/dl)	83.4 (71.0–91.9)	123.6 (113.1–140.2) ^*^	127.4 (113.1–146.3) ^*^	87.6 (77.6–95.0)^*†‡^
HDL-cholesterol (mg/dl)	48.6 (42.1–56.4)	50.6 (45.2–57.5) ^*^	45.8 (40.9–52.1) ^*†^	39.4 (34.4–44.4) ^*†‡^
Non-HDL-cholesterol (mg/dl)	97.7 (88.4–106.2)	135.1 (123.2–151.0) ^*^	157.5 (140.7–178.8) ^*†^	121.6 (108.1–138.2) ^*†‡^

Data are medians (interquartile range)

^*^ *P* < 0.008 compared with individuals with normal-TG normal-LDL-C

^†^ *P* < 0.008 compared with individuals with hyper-TG normal-LDL-C

^‡^ *P* < 0.008 compared with individuals with normal-TG hyper-LDL-C

**Table 2:** Characteristics of participants by triglycerides (TG) and non-HDL-cholesterol status

	Normal-TG normal-non-HDL-C	Hyper-TG normal- non-HDL-C	Normal-TG hyper- non-HDL-C	Hyper-TG hyper- non-HDL-C
n	1153	484	894	1178
Age (years)	48.0 (40.0–55.0)	54.0 (46.0–63.0) ^*^	51.0 (42.0–58.0) ^*^	50.0 (41.0–59.0)^†^
Body mass index (kg/m^2^)	25.5 (24.1–27.2)	25.6 (24.1–27.5)	26.0 (24.3–27.7)	25.2 (23.7–27.1)^‡^
Systolic blood pressure (mmHg)	122.0 (117.0–134.0)	128.0 (120.0–138.0)^*^	130.0 (120.0–140.0)^*^	123.0 (116.0–135.0)^†‡^
Diastolic blood pressure (mmHg)	80.0 (74.0–90.0)	80.0 (75.0–90.0)	80.0 (77.0–90.0)	80.0 (70.0–87.0)
Fasting plasma glucose(mmol/l)	5.1 (4.8–5.5)	5.2 (4.8–5.6)	5.2 (4.8–5.7)	5.1 (4.7–5.5)
Uric acid (mmol/l)	5.3 (4.6–6.3)	5.9 (5.2–6.7)^*^	5.9 (5.2–6.9)^*^	5.4 (4.6–6.2)^†‡^
Alanine aminotransferase (U/L)	22.0 (16.0–32.0)	25.0 (18.0–35.0)^*^	29.0 (20.0–43.0)^*^	29.0 (20.0–41.0)^*†^
Total cholesterol (mg/dl)	158.3 (145.2–167.2)	201.2 (188.4–217.4)^*^	205.4 (188.0–227.8)^*^	159.8 (147.1–171.0)^*†‡^
Triglycerides (mg/dl)	94.7 (70.8–117.7)	112.4 (92.0–130.1)^*^	221.2 (181.4–292.9)^*†^	202.7 (172.6–255.8)^*†‡^
LDL-cholesterol (mg/dl)	88.0 (74.5–99.6)	135.5 (124.3–149.4)^*^	124.7 (107.3–145.2)^*†^	99.6 (87.3–111.6)^*†‡^
HDL-cholesterol (mg/dl)	40.5 (35.5–45.6)	51.4 (45.2–59.1)^*^	45.2 (39.8–51.4)^*†^	49.0 (43.2–56.4)^*†‡^
Non-HDL-cholesterol (mg/dl)	110.2 (96.9–120.5)	146.7 (137.1–162.5)^*^	158.9 (143.6–180.3)^*†^	117.8 (106.2–123.2)^*†‡^

Data are medians (interquartile range)

^*^ *P* < 0.008 compared with individuals with normal-TG normal-non-HDL-C

^†^ *P* < 0.008 compared with individuals with hyper-TG normal-non-HDL-C

^‡^ *P* < 0.008 compared with individuals with normal-TG hyper-non-HDL-C

When using non-HDL-C and LDL-C to identify patients with abnormal LDL, discordant classifications occurred for 18.3% of participants who had an LDL-C ≥ 100 mg/dl and non-HDL-C < 130 mg/dl, and for 5.6% who had an non-HDL-C ≥ 130 mg/dl and LDL-C < 100 mg/dl.

**Fig. 3:**
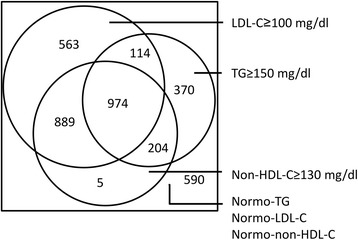
Venn diagram for a visual display of how the three parameters (triglycerides, LDL-cholesterol, and non-HDL-cholesterol) cluster together

The correlations of non-HDL-C with LDL-C according to NAFLD status and TG levels were displayed in Table 3. In the NAFLD state, the correlation between non-HDL-C and LDL-C is similar in the hypertriglyceridemic (*r* = 0.561, *P* < 0.01) and normotriglyceridemic subgroups (*r* = 0.553, P < 0.01). We then evaluated the discordance between classifications based on LDL-C and non-HDL-C according to TG levels (TG < 150 mg/dl and TG ≥ 150 mg/dl) (Fig. 3). The concordance between non-HDL-C- and LDL-C-based diagnoses of abnormal LDL was moderate in both the hypertriglyceridemic (ҝ = 0.56; 95% CI 0.52–0.60) and normotriglyceridemic (ҝ = 0.47; 95% CI 0.44–0.51) subgroups. Non-HDL-C detected fewer patients with abnormal LDL than LDL-C in normotriglyceridemic patients: 563 of the 2047 patients considered as abnormal LDL according to LDL-C fell into the normonon-HDL-C phenotype, whereas only 5 of the 2047 patients considered as abnormal LDL according to non-HDL-C fell into the normoLDL-C phenotype (Fig. 3). However, non-HDL-C detected more patients with abnormal LDL than LDL-C in hypertriglyceridemic patients: 114 of the 1662 patients considered as abnormal LDL according to LDL-C fell into the normonon-HDL-C phenotype, whereas 204 of the 1662 patients considered as abnormal LDL according to non-HDL-C fell into the normoLDL-C phenotype.

**Table 3:** Correlations between non-HDL-C and LDL-C according to nonalcoholic fatty liver disease (NAFLD) status and triglycerides levels

	Without NAFLD	NAFLD
Normotriglyceridemia	0.554	0.553
Hypertriglyceridemia	0.616	0.561

## Discussion

To our knowledge, this is the first study to report a comparison between LDL-C and non-HDL-C for the classification of patients with NAFLD into dyslipidemic phenotypes.

We noted that the prevalence of discordance defined according to LDL-C and non-HDL-C cut points was common, reaching 23.9%. Although fewer proportions of abnormal LDL were identified by non-HDL-C, non-HDL-C identified high-risk phenotypes that are not detected by standard lipid profile in hypertriglyceridemic patients, indicating that non-HDL-C identifies patients at risk better than LDL-C in hypertriglyceridemic patients.

Although LDL-C and non-HDL-C are closely correlated, they assess different elements of lipid metabolism. LDL-C is the amount of cholesterol contained in LDL particles, whereas non-HDL-C is the total amount of cholesterol carried by LDL, IDL, and VLDL particles. Mechanistically, the cholesterol content within LDL particles can vary substantially as cholesterol ester within LDL particles can be exchanged for triglyceride molecules within VLDL particles [20]. The considerable discordance between LDL-C- and non-HDL-C-based identification of dyslipidemia phenotypes supported this notion. In the hypertriglyceridemic state, a triglyceride molecule from VLDL particles is exchanged for a cholesterol ester in LDL particles, producing relatively triglyceride-enriched LDL particles and relatively cholesterol-enriched VLDL particles [20]. Hence, LDL-C underestimates the concentrations of non-HDL-C in the setting of hypertriglyceridemia.

It is already established that hypertriglyceridemia, which was typically observed in NAFLD, accounted for the hepatic over-production of VLDL particles and the increased VLDL size [21]. The increased VLDL particles and VLDL size prevent lipoprotein lipase-mediated clearance of triglyceride molecules within VLDL, thereby producing triglyceride-rich lipoprotein remnants. Hepatic lipases can hydrolyze these remnant particles, producing small dense lipoprotein particles and imparting increased CVD risk, suggesting that normal LDL-C but hyper- non-HDL-C generally reflects increased concentrations of smaller, cholesterol-depleted LDL particles among those with hypertriglyceridemia. All these support the notion that the dysregulation of cholesterol played a vital role in the pathogenesis of NAFLD [8, 22]. A recent prospective study observed that the increased non-HDL-C levels precedes the onset of NAFLD [9], which further highlighted a causal relationship between the impairment of cholesterol regulation and the NAFLD onset.

In the present study, non-HDL-C identifies a subgroup of patients with normoLDL-C who had hyper-non-HDL-C. The identification of this subgroup is noteworthy as the core lipid composition of LDL is altered in a proatherogenic direction. Multiple mechanistic, observational and experimental trials have shown that alterations in VLDL, LDL and IDL synthesis and release may play a role in the pathogenesis of NAFLD [23] and explain the observed increased CVD risk among those with NAFLD. Emerging evidence revealed that cholesterol-lowering therapy was effective in reducing CVD events and improving liver damage among those with NAFLD [4, 24]. Hence, detection and treatment of dyslipidemia by incorporating non-HDL-C is therefore of importance among patients with NAFLD and for preventing and treating CVD, especially in hypertriglyceridemic patients. Furthermore, accumulating prospective studies indicate the superiority of non-HDL-C over LDL-C in predicting CVD [11, 12]. Our present data supports the currently applicable guidelines that recommend non-HDL-C as alternative targets of therapy to LDL-C for the management of dyslipidemias in individuals with hypertriglyceridemia [18, 25].

The present study has several limitations. First, lipoproteins were not measured by the more sophisticated method nuclear magnetic resonance spectroscopy. However, increasing evidence suggested that the association of coronary artery calcification with nuclear magnetic resonance-measured lipoproteins was comparable to that with standard lipids [26]. Second, we studied a cohort of Chinese patients with NAFLD, thus, the present results may not be generalizable to other racial or ethnic patients. Third, the cross-sectional nature of this study makes it difficult to infer causality between different lipid phenotypes and the relative CVD risk among patients with NAFLD. Nevertheless, the analysis of the dyslipidemic classification based on LDL-related measures was not influenced by this particular design. At last, NAFLD was diagnosed by ultrasonography, which is a reasonably accurate technique for detecting modest amounts of liver fat (>30% liver fat in filtration), participants with minor amounts of fatty infiltration might not have been captured.

In conclusion, among patients with NAFLD, LDL-C is superior to non-HDL-C in the detection of high-risk phenotypes in normotriglyceridemic patients, whereas non-HDL-C seems to be superior in hypertriglyceridemic patients. Our findings together with the logistical advantages of non-HDL-C (a cost-free test, and can provide an important value in CVD risk stratification) may support it as a first-line component to be evaluated in dyslipidemic classification and for diagnostic and even therapeutic purposes among those with NAFLD in the setting of hypertriglyceridemia.

## Abbreviations

ACC: American College of Cardiology Foundation
ADA: American Diabetes Association
BMI: body mass index
BP: blood pressure
CVD: cardiovascular disease
HDL-C: high-density lipoprotein cholesterol
IDL: intermediate-density lipoprotein
IQR: interquartile range
LDL-C: low-density lipoprotein-cholesterol
NAFLD: nonalcoholic fatty liver disease
non-HDL-C: non-high-density lipoprotein cholesterol
TC: total cholesterol
TG: triglyceride
VLDL: very-low-density lipoprotein
WHO: World Health Organization
WISCO: Wuhan Iron and Steel Company
